# Professional Prospects

**Published:** 1900-09-08

**Authors:** 


### The Hospital

A Journal of

The Medical Sciences and Hospital Administration.

Vol. XXVIII.—No. 728.]

Edited by Sir Henry Burdett, K.C.B., and Solomon C. Smith, M.D., M.R.C.P.

[September 8, 1900.

### Professional Prospects.

There is much reason for believing that the immediate prospects before those entering the medical profession are brighter than they have been for some time past. The action of the General Medical Council in putting a stop, so far as it has been able so to do, to the employment of unqualified assistants in professional capacities, and the consequent increase in the demand for assistants and junior partners which we are assured has taken place, make it probable that, at least in the junior ranks of the profession, a better scale of remuneration will be obtainable than used to be the case a few years ago. Thus the temptation is held before those who are hovering on the brink of a medical career that, once they become qualified, they may fairly hope, if their conduct be good, to become almost immediately self-supporting. This is more than could be said some years ago, and this prospect of "quick returns" is sure to prove attractive to many who possess capital enough, or, perhaps, to speak more correctly, whose fathers have capital enough, to pay for their medical education, but who see no likelihood of having much beyond.

At the same time, it must be remembered that these brighter prospects at the opening of the qualified practitioner's career are to a large extent bought at the expense of those who have already become established, and that the greater demand for assistants, and the higher salaries they are able to obtain, do not arise from "increase of business," to put the matter in commercial language. That there is some increase in the demand for medical services is probable enough. With extending education, and with the growing tendency to recognise specialism in every department of work, there is a tendency to run for the doctor more often than used to be the case. In everything we tend more and more to use special tools and special workmen for special work, and we need hardly wonder that people who hesitate to sew on their own buttons, for fear they should not do it right, send for the doctor in many circumstances in which their grandmothers would have been content with some antiquated household remedy, and that there may thus be some increase in the demand for medical services. On the other hand, there is a constant lowering of professional remuneration, and an untold increase of gratuitous work. We imagine then that practitioners throughout the country will agree that it is out of their pockets, and not out of the pockets of the public, that the higher salaries of their assistants come, and that bright and hopeful as the outlook may seem to newly-qualified men seeking posts as assistants, their prospects as practitioners are anything but rosy. It used to be thought that the newly-fledged practitioner had before him a weary time of waiting before he could earn bread and butter, but that when once on his feet all was well. Now, however, things are very different. There is immediate employment for most men, and the assistant earns kid gloves as well as bread and butter. Indeed, he becomes a much smarter man than his master. It is later on that the trouble comes, and many a practitioner might, we fear, truthfully confess that at the end of the year's work he had put by far less than his assistant could have done if he had lived with equal thrift.

On the whole, then, it would appear that the monetary attraction of the profession is not great. The man of strongly developed business instincts who can "work up" a practice, who is ready to "push" whatever side of his work pays best, who "invests" in a carriage not when he wants it but when he thinks it will "pay," and who has a good idea of "value" all round, very likely does well. So he would have done, and perhaps more quickly, in any trade. But the ultimate prospects of the man who regards his profession as an interesting scientific pursuit, who prides himself chiefly on good work well done, who hates the mercantile side of his daily round, who dislikes above all things asking for payment, who would shiver at the idea of suing in the County Court, and who feels degraded when his patient in a cheery voice says, "Well, doctor, and how much for that job?"—just as if he were a yard of tape or a pound of candles; to such a man, and there are hundreds of such, the profession is apt to become a woebegone series of disappointments. Some men have good fortune—or, perhaps, it would be more correct to say that some men have the business aptitude to see and to snatch at the hand of fortune when it is offered—and these lift themselves out of the rut, become consultants, great surgeons, and what not; have their country houses and their shooting, and bustle it with the best. But many of those who are forced to spend their lives in general practice have a hard time. Some even of these undoubtedly succeed, as they might have done still more markedly on the Stock Exchange or elsewhere. Others fail, and the worst of it is that, so far as the professional and scientific point of view is concerned, it may often be truly said that while success is no credit, failure is no disgrace, which hardly agrees with the picture so commonly drawn by the authors of those interesting but often delusive orations called "Introductory Addresses."

